# Real-time single-molecule imaging in zebrafish embryos uncovers noncanonical translation

**DOI:** 10.1126/sciadv.aeb6883

**Published:** 2026-10-07

**Authors:** Maëlle Bellec, Jie Liang, Kenny Mattonet, Tatsuya Morisaki, Margaux Lay, Hans-Martin Maischein, Damien Avinens, Vincent Martinet, Delphine Muriaux, Timothy J. Stasevich, Jérémy Dufourt, Didier Y. R. Stainier

**Affiliations:** ^1^ Department of Developmental Genetics, Max Planck Institute for Heart and Lung Research, Bad Nauheim, Hessen 61231, Germany.; ^2^ Imaging Platform, Max Planck Institute for Heart and Lung Research, Bad Nauheim, Hessen 61231, Germany.; ^3^ Department of Biochemistry and Molecular Biology, Colorado State University, Fort Collins, CO, USA.; ^4^ Institut de Recherche en Infectiologie de Montpellier, CNRS UMR 9004, Université de Montpellier, 1919 Route de Mende, Montpellier 34293, Cedex 5, France.; ^5^ German Centre for Cardiovascular Research (DZHK), partner site Rhine-Main, Bad Nauheim, Germany.; ^6^ Cardio-Pulmonary Institute (CPI), Bad Nauheim, Germany.

## Abstract

Precise spatiotemporal control of protein synthesis is essential during embryogenesis, yet directly measuring translation kinetics in vivo remains challenging in vertebrates. In particular, it remains unclear how translation efficiency is determined for key developmental regulators and which kinetic steps limit their production. Here, we used ALFA array–based nascent chain labeling combined with lattice light-sheet microscopy to visualize *bmp2b* translation in real time and at single-molecule resolution in early zebrafish embryos. When combined with MS2/MCP labeling to visualize all *bmp2b* messenger RNA (mRNAs), we found that only some of them are being actively translated, suggesting that limited mRNA translation competence contributes to overall translation efficiency. We found that *bmp2b* translation operates below a maximal initiation regime, as replacement of its untranslated regions (UTRs) with viral UTRs increases ribosome loading. Furthermore, the *bmp2b*, but not *actb2*, 5′UTR supports cap-independent translation with ribosome loading comparable to cap-dependent initiation. Together, this approach provides a quantitative in vivo framework to dissect translation kinetics during early vertebrate development.

## INTRODUCTION

The execution of complex developmental programs relies on the precise regulation of gene expression in both space and time. While transcriptional profiling has provided valuable insight into mRNA abundance, it is increasingly recognized that mRNA levels do not always reflect protein output, especially during rapid cell state transitions such as those observed during embryogenesis (*1*–*3*). These observations highlight the importance of studying gene expression as a dynamic and multilayered process, where regulation extends beyond transcription to encompass mRNA localization, translation, and degradation.

Among these processes, translational control provides a rapid and flexible mechanism to modulate protein production without altering transcript abundance. By adjusting the efficiency and timing of protein synthesis, translation can fine-tune gene expression dynamics during developmental processes such as cell fate specification and tissue patterning, as well as during regeneration (*3*–*10*). This level of regulation is especially relevant for morphogens whose spatial concentration gradients must be tightly controlled to ensure robust pattern formation (*8*, *11*, *12*). In zebrafish, the bone morphogenetic protein gene *bmp2b* encodes a central morphogen that establishes the dorsoventral axis and controls gastrulation (*13*–*16*). *bmp2b* transcripts are distributed in a ventral-to-dorsal gradient (*17*), yet unexpectedly little is known about how their translation is regulated. Notably, despite Bmp2b’s critical role in early development, Bmp2b protein levels are low or absent from proteomic datasets (*18*, *19*), and ribosome profiling indicates low translation efficiency (*20*). These observations raise an intriguing question: How are Bmp2b protein levels quantitatively controlled to generate a robust morphogen gradient during early development?

One possibility is that translation of *bmp2b* mRNAs is regulated at multiple levels, including the fraction of mRNAs engaged in translation, as well as ribosome initiation and elongation rates. However, the real-time dynamics of *bmp2b* translation in vivo remain largely unexplored, limiting our understanding of how morphogen production is quantitatively controlled during early vertebrate development.

To investigate how Bmp2b translation is regulated, it is essential to consider the regulatory elements that influence translation, especially the 5′ cap, the polyadenylate [poly(A)] tail and untranslated regions (UTRs) of mRNAs. The 5′ cap promotes 40*S* ribosome recruitment and canonical initiation (*21*–*23*), while 5′UTRs contain structures and motifs that modulate the initiation and efficiency of translation (*24*–*27*). A notable example of such regulation is provided by viruses, such as the 5′UTR of the *Orthoflavivirus* Zika virus (ZIKV) RNA, which contains highly structured elements and has been shown to exhibit internal ribosome entry site (IRES) activity in cell culture (*28*, *29*). This IRES activity enables cap-independent translation, allowing viral protein synthesis to proceed even when host cap-dependent translation is compromised (*28*, *29*). Moreover, ZIKV can infect zebrafish (*30*), offering a unique opportunity to study these noncanonical translational mechanisms in a live vertebrate model. Although IRES-mediated and, more broadly, cap-independent translation are well characterized in vitro and in viral systems, their role in vertebrate genes remains poorly understood. This lack of knowledge is largely due to the complexity of in vivo regulatory environments and the technical challenges of monitoring translation dynamics with high spatial and temporal resolution in developing organisms.

Efforts to dissect translation dynamics in vivo have long been constrained by the limited availability of tools that can monitor translation at high resolution in living organisms. Antibody-based nascent chain imaging approaches have emerged as powerful strategies to visualize translation at the single-cell level (*31*–*36*) and, more recently, have been successfully adapted to *Drosophila* embryos (*37*–*41*). However, their application in vertebrate systems remains at an early stage. Technical challenges, including context-dependent variability in protein stability, heterogeneous expression, and limitations in imaging depth, continue to hinder their widespread implementation in complex tissues (*36*, *42*). Overcoming these challenges in zebrafish embryos would represent a substantial advance, enabling the study of translation in a model that offers greater spatial and developmental complexity than cell culture or invertebrate systems.

Here, we deploy the ALFA_array system to achieve real-time imaging of *bmp2b* translation in live zebrafish embryos, making the first application of this technology in a vertebrate system. We first generated stable transgenic lines expressing a nanobody-based reporter for nascent peptide detection and validated the system using synthetic mRNA and plasmid injections. Using lattice light-sheet microscopy (LLSM) (*43*), we tracked individual *bmp2b* polysomes with subcellular resolution. We then generated a stable transgenic line expressing tagged *bmp2b* to quantify its native translation dynamics and estimated its kinetic parameters. Furthermore, by exploiting the unique properties of ZIKV UTRs, we were able to probe both cap-dependent and cap-independent mechanisms in a physiological context. Together, this work expands the translational imaging toolbox for vertebrate embryos and provides a framework to quantitatively study mRNA translation in vivo, including the respective contributions of translation competence, ribosome initiation rates, and UTR-dependent regulatory mechanisms.

## RESULTS

### Implementation of the ALFA_array system for in vivo monitoring of translation

To establish a live imaging approach for visualizing translation in zebrafish embryos using antibody-based detection of nascent peptides, we first adapted the well-established SunTag system (*31*, *32*, *34*, *35*), a gold standard for monitoring translation dynamics in live cells and *Drosophila* embryos through single-chain variable fragment (scFv) recognition of epitope-tagged nascent chains. We generated a transgenic zebrafish line expressing an anti-suntag scFv fused to monomeric superfolder green fluorescent protein 2 (GFP2) (scFv-msGFP2) (*44*), a fluorescent protein previously shown to display high brightness and monomericity in *Drosophila* (*38*), under the control of a ubiquitin promoter (*45*). Despite successful transgene expression, we observed prominent fluorescent aggregates beginning at 8 hours postfertilization (fig. S1A), limiting the utility of this system for live imaging of translation in zebrafish.

To overcome the limitations encountered with the SunTag system, we next implemented an orthogonal approach, originally developed in *Drosophila* embryos and mammalian cells, the ALFA_array system (*37*) (Fig. 1A). This bipartite strategy comprises multiple copies of the ALFA-tag (*46*) inserted into the gene of interest and specifically recognized with high affinity (*46*) by a fluorescently labeled anti–ALFA-tag nanobody (NB-ALFA). To enable broad applicability of this method in zebrafish, we generated a transgenic line that ubiquitously expresses an optimized NB-ALFA fused to msGFP2 (see Materials and Methods) (Fig. 1A).

**Fig. 1.**
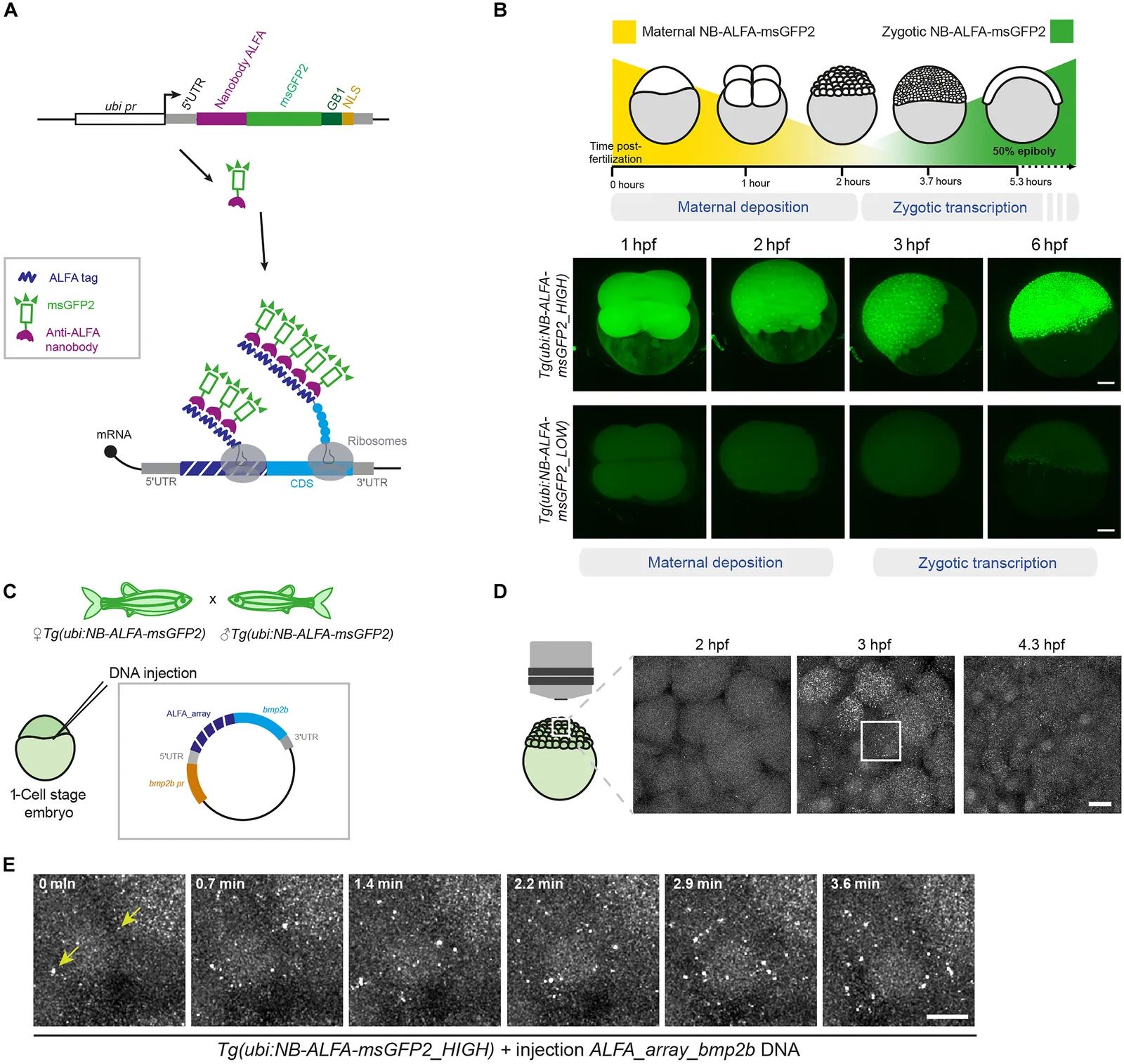
Implementation of the ALFA_array system in zebrafish embryos for live imaging of translation. (**A**) Schematic of the ALFA_array system for live imaging of translation. Top: Schematic of the construct used to generate transgenic lines expressing NB-ALFA-msGFP2 under the control of a ubiquitin promoter. Upon translation, ALFA tags (dark blue) inserted into the gene of interest (blue, CDS: Coding sequence) become bound by the anti–ALFA-tag nanobody (NB-ALFA) fused to a fluorescent protein (green). GB1, immunoglobulin-binding protein G, B1 domain; NLS, nuclear localization signal. (**B**) Schematic representation of the maternal-to-zygotic transition in zebrafish with the maternal deposition of NB-ALFA-msGFP2 in yellow and the zygotic NB-ALFA-msGFP2 expression in green. Bottom: Maximum intensity–projected images of light-sheet movies of *Tg(ubi:NB-ALFA-msGFP2_HIGH)* and *Tg(ubi:NB-ALFA-msGFP2_LOW)* embryos with maternal and zygotic expression of the anti-ALFA nanobody. (**C**) Schematic of the cross used to live image translation in zebrafish embryos as shown in (D). Bottom: Schematic of the plasmid containing *bmp2b* fused to the ALFA_array tag, which was injected into one-cell stage *Tg(ubi:NB-ALFA-msGFP2_HIGH)* embryos. (**D**) Schematic of the experimental setup to live image translation in a zebrafish embryo (left) using confocal microscopy. Maximum intensity–projected images from a confocal movie of a *Tg(ubi:NB-ALFA-msGFP2_HIGH)* embryo injected with *ALFA_array_bmp2b* DNA (right). White square represents the zoomed area for the images shown in (E). (**E**) Zoomed in images from the confocal movie in (D). Yellow arrows point to translation dots. Scale bars, 100 μm (B), 20 μm (D), and 10 μm (E). hpf, hours postfertilization.

We first observed different expression levels of the NB-ALFA-msGFP2 transgene in the F_1_ generation (fig. S1, B and C) and established two lines, one with “low expression” [*Tg(ubi:NB-ALFA-msGFP2_LOW)*] and one with “high expression” [*Tg(ubi:NB-ALFA-msGFP2_HIGH)*], the latter showing a ∼2.5-fold increase in expression compared with the former (fig. S1D). Next, by using light-sheet microscopy, we confirmed that NB-ALFA-msGFP2 was maternally deposited (Fig. 1B and movie S1) and started to be zygotically expressed at around 4 hours postfertilization (fig. S1, E and F, and movie S1). Light-sheet and confocal imaging of these reporter lines revealed diffuse and uniform fluorescence during early development, with no evidence of aggregation (Fig. 1B and fig. S1G), thereby enabling their use for high-resolution live imaging of translation.

To investigate the translation dynamics of a key developmental gene, we applied the ALFA_array system to the morphogen Bmp2b. We inserted an array of 32× ALFA-tags at the N terminus of Bmp2b and injected in vitro–transcribed mRNA into one-cell stage wild-type (WT) embryos (fig. S2A). Western blot analysis detected a predominant band at ∼115 kDa, corresponding to the molecular weight expected for the full-length fusion protein (ALFA_array_Bmp2b) (fig. S2B); it also revealed ALFA_array_Bmp2b expression as early as 1.5 hours postfertilization, followed by a substantial decrease by 6 hours postfertilization (fig. S2B). No signal was detected in embryos injected with *bmp2b* mRNA lacking the ALFA_array, thereby confirming the specificity and low background of the detection system (fig. S2B). To better mimic endogenous regulation, we next injected a plasmid containing the 2.27-kb *bmp2b* promoter (*16*) and intronic sequences (fig. S2C), which drove expression of ALFA-tagged Bmp2b from ∼4.3 hours postfertilization, with levels increasing at least until 12 hours postfertilization (fig. S2D). This expression reflects the temporal pattern of endogenous *bmp2b* mRNA (*47*) (fig. S2E) and is consistent with ribosome profiling data (*48*) (fig. S2F).

To determine whether the *bmp2b-ALFA-array* construct retains biological activity and whether NB-ALFA binding interferes with its function, we performed functional assays in vivo. We injected the *bmp2b-ALFA-array* construct into both WT embryos and embryos expressing NB-ALFA-msGFP2 and compared the resulting phenotypes with those obtained after injection of nontagged *bmp2b* mRNA, which is well known to induce ventralization (*16*, *49*, *50*). In both genetic backgrounds, injection of the *bmp2b-ALFA-array* construct into one-cell stage embryos produced ventralization phenotypes comparable to those observed in embryos injected with the nontagged *bmp2b* control (fig. S3A), indicating that the ALFA_array does not abolish Bmp2b function and that NB-ALFA binding does not measurably affect its biological activity.

To further test the binding properties of NB-ALFA in zebrafish embryos, we performed fluorescence recovery after photobleaching (FRAP) in *Tg(ubi:NB-ALFA-msGFP2_HIGH)* embryos injected with an *H2B_mCherry_ALFA-tag* plasmid (*51*) (fig. S2G). We observed minimal recovery of NB-ALFA-msGFP2 fluorescence over a 4.5-min period (fig. S2, G and H), consistent with strong binding of the nanobody to the ALFA-tag. This finding aligns with previously published data showing slow recovery kinetics of histone 2B (H2B), which typically occurs over hours (*51*, *52*). In contrast, FRAP analysis in embryos expressing only NB-ALFA-msGFP2 (i.e., without an ALFA-tagged target) revealed rapid recovery, consistent with the free diffusion of the unbound nanobody as well as the absence of visible aggregates (fig. S2I).

Next, to visualize translation in zebrafish embryos, we initially performed conventional live confocal imaging on *Tg(ubi:NB-ALFA-msGFP2_HIGH)* embryos injected with the ALFA_array_*bmp2b* plasmid (Fig. 1C). From ∼2.5 hours postfertilization, we observed the emergence of discrete fluorescent foci in a mosaic pattern (Fig. 1D), consistent with specific translation events. We observed that these puncta were motile, likely reflecting active sites of *bmp2b* translation (Fig. 1E).

In summary, we have established transgenic zebrafish lines that ubiquitously express NB-ALFA throughout development, providing a robust, aggregation-free platform for antibody-based detection of nascent peptides in vivo. Using the *ALFA_array_bmp2b* reporter, we directly visualize active *bmp2b* translation in early zebrafish embryos by detecting the stable binding of NB-ALFA-msGFP2 to ALFA-tagged nascent chains, an essential requirement for accurate and quantitative analysis of mRNA translation dynamics.

### Real-time visualization of *bmp2b* translation dynamics on single mRNAs in zebrafish embryos using LLSM

Although translation sites were clearly detectable with confocal imaging, the temporal resolution was insufficient for detailed kinetic analysis, and the translation spots could not be resolved at higher frame rates. To overcome these limitations, we used LLSM for high-resolution, rapid volumetric imaging with minimal phototoxicity (Fig. 2A). Using this imaging technique, we achieved volumetric imaging over an area of ∼300 × 100 μm with a depth of 35 μm at a rate of one stack per second, enabling real-time tracking of translation foci over several minutes across multiple cells simultaneously (Fig. 2, B and C, and movie S2). We detected bright foci, which were absent in cells without *ALFA_array_bmp2b* expression, likely corresponding to translation sites (polysomes) (fig. S3B). To test whether these foci indeed represented active *bmp2b* translation, we performed control injections with either *ALFA_array_bmp2b* mRNA, *ALFA_array_bmp2b* mRNA together with the translation inhibitor puromycin, or *bmp2b* mRNA lacking the ALFA_array. Fluorescent foci were completely absent in both puromycin-treated embryos and ALFA-less conditions (Fig. 2D and movie S3), implying that the observed signal arises from active translation sites.

**Fig. 2.**
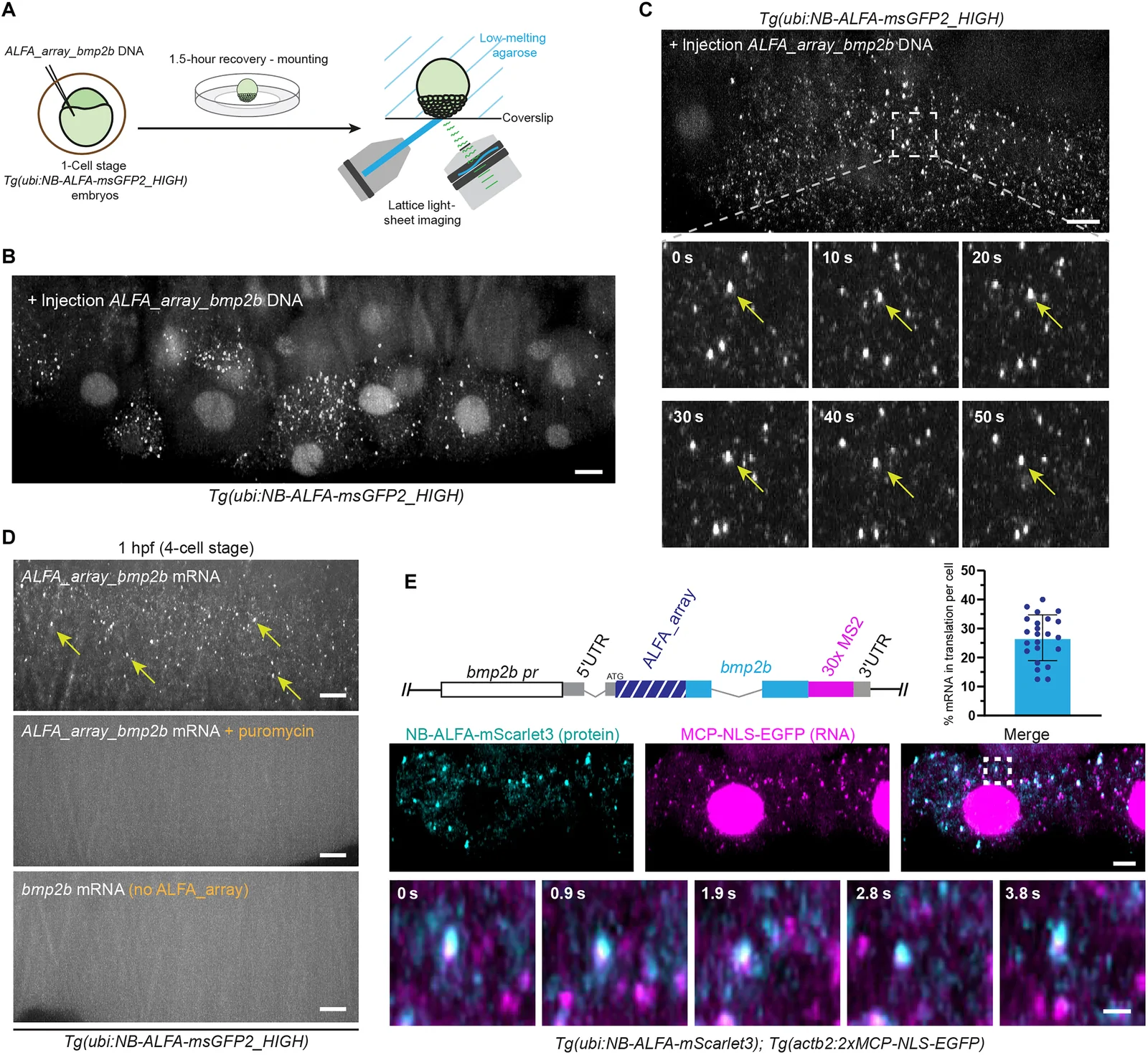
Visualization and validation of *bmp2b* translation in zebrafish embryos using LLSM. (**A**) Schematic of experimental setup for LLS imaging of zebrafish embryos. Embryos were injected at the one-cell stage, manually dechorionated, and then mounted in low–melting point agarose in a glass-bottom dish with the animal pole oriented toward the coverslip. (**B**) Maximum intensity–projected image from an LLS movie of a ∼4.3-hours postfertilization *Tg(ubi:NB-ALFA-msGFP2_HIGH)* embryo injected with *ALFA_array_bmp2b* DNA. (**C**) Maximum intensity–projected image from an LLS movie of a ∼4.3-hours postfertilization *Tg(ubi:NB-ALFA-msGFP2_HIGH)* embryo injected with *ALFA_array_bmp2b* DNA and zoomed-in images from the movie showing the translation dots of *ALFA_array_bmp2b* (arrow) over time. (**D**) Representative maximum intensity–projected images from LLS movies of 1-hours postfertilization *Tg(ubi:NB-ALFA-msGFP2_HIGH)* embryos injected with *ALFA_array_bmp2b* mRNA without or with puromycin or with *bmp2b* mRNA (no ALFA_array). Yellow arrows point to translation dots, absent in the presence of puromycin or when embryos were injected with the construct lacking the *ALFA_array*. (**E**) Schematic of the *bmp2b* construct containing the *ALFA_array* and 30x MS2. Representative image from an LLS movie of a ∼4.3-hours postfertilization *Tg(ubi:NB-ALFA-mScarlet3); Tg(actb2:2xMCP-NLS-EGFP)* embryo injected with *ALFA_array_bmp2b_30xMS2* DNA. MCP-EGFP is represented in magenta and corresponds to mRNA molecules (nuclear accumulation is due to the presence of NLS), and NB-ALFA-mScarlet3 (nascent translation) is represented in cyan. The histogram shows the quantification of the percentage of mRNA molecules in translation (colocalized MCP-NLS-EGFP and NB-ALFA-mScarlet3 signals). Scale bars, 10 μm [(B) to (E)], 5 μm (E), and 1 μm [zoomed in (E)].

We first validated the ALFA_array system in fixed embryos, using a mosaic expression approach through plasmid injection, with hybridization chain reaction fluorescence in situ hybridization (HCR-FISH) for ALFA-tagged *bmp2b* mRNA and immunostaining for the ALFA-tag epitopes. We observed clear coexpression (fig. S3C), thereby demonstrating specificity and active translation of the mRNAs generated from the injected plasmid. However, this approach in fixed embryos fails to achieve reliable single-molecule resolution, thereby preventing quantitative measurement of translation of individual mRNAs or the kinetic analysis of Bmp2b protein output.

To overcome this limitation, we next aimed to simultaneously visualize single mRNAs and their translation in live. To this end, we combined the ALFA_array system with the MS2/MCP system (*53*) by generating a construct containing *ALFA_array_bmp2b* and 30 MS2 loops in the 3′UTR of *bmp2b* (Fig. 2E). We then established a zebrafish line stably expressing the nanobody fused to mScarlet3, crossed it with the *Tg(actb2:2xMCP-NLS-EGFP)* line (*54*), and injected into the resulting progeny the tagged *bmp2b* construct. This strategy, together with the high temporal resolution of LLSM (180 ms per frame), allowed us to visualize putative transcription sites in nuclei, as identified by their larger size, higher fluorescence intensity, and more restricted movement compared with individual mRNA molecules (*37*, *54*) or individual mRNA molecules in motion (fig. S3D). We could also monitor translation occurring on these single mRNA molecules (Fig. 2E and movies S4 and S5) and found that ∼26% of mRNAs were actively translating (Fig. 2E). Consistent with reports that ∼20 to 60% of mRNAs from various genes are being actively translated in live vertebrate cells, our data place *bmp2b* within the modestly translated range characteristic of regulated transcripts (*32*, *34*).

### Stable insertion of an ALFA_array-tagged *bmp2b* transgene enables in vivo quantification of translation kinetics in developing zebrafish embryos

To analyze translation dynamics in a more physiologically relevant context, we generated a stable transgenic zebrafish line expressing *bmp2b* tagged with the ALFA_array and under the control of the 2,27-kb *bmp2b* promoter [*Tg(bmp2b:ALFA_array_bmp2b)*] (Fig. 3A). This line recapitulated the expression pattern reported for the proximal 2,27-kb *bmp2b* promoter (*16*, *55*), exhibiting broad expression at 4.3 hours postfertilization and restricted expression in the dorsal organizer and to a lesser extent on the ventral side by shield stage (6 hours postfertilization) (Fig. 3B). We further confirmed the specificity of the translation foci by their absence outside the *bmp2b* transgene expression domain (fig. S4A). Time-lapse imaging of *bmp2b* translation from 4.3 to 6.5 hours postfertilization revealed a pattern initially broad across the embryo and later restricted to one side (fig. S4B). We also observed *bmp2b* mRNA translation in the developing eye at 24 hours postfertilization using lattice light-sheet (LLS) imaging (fig. S4C).

**Fig. 3.**
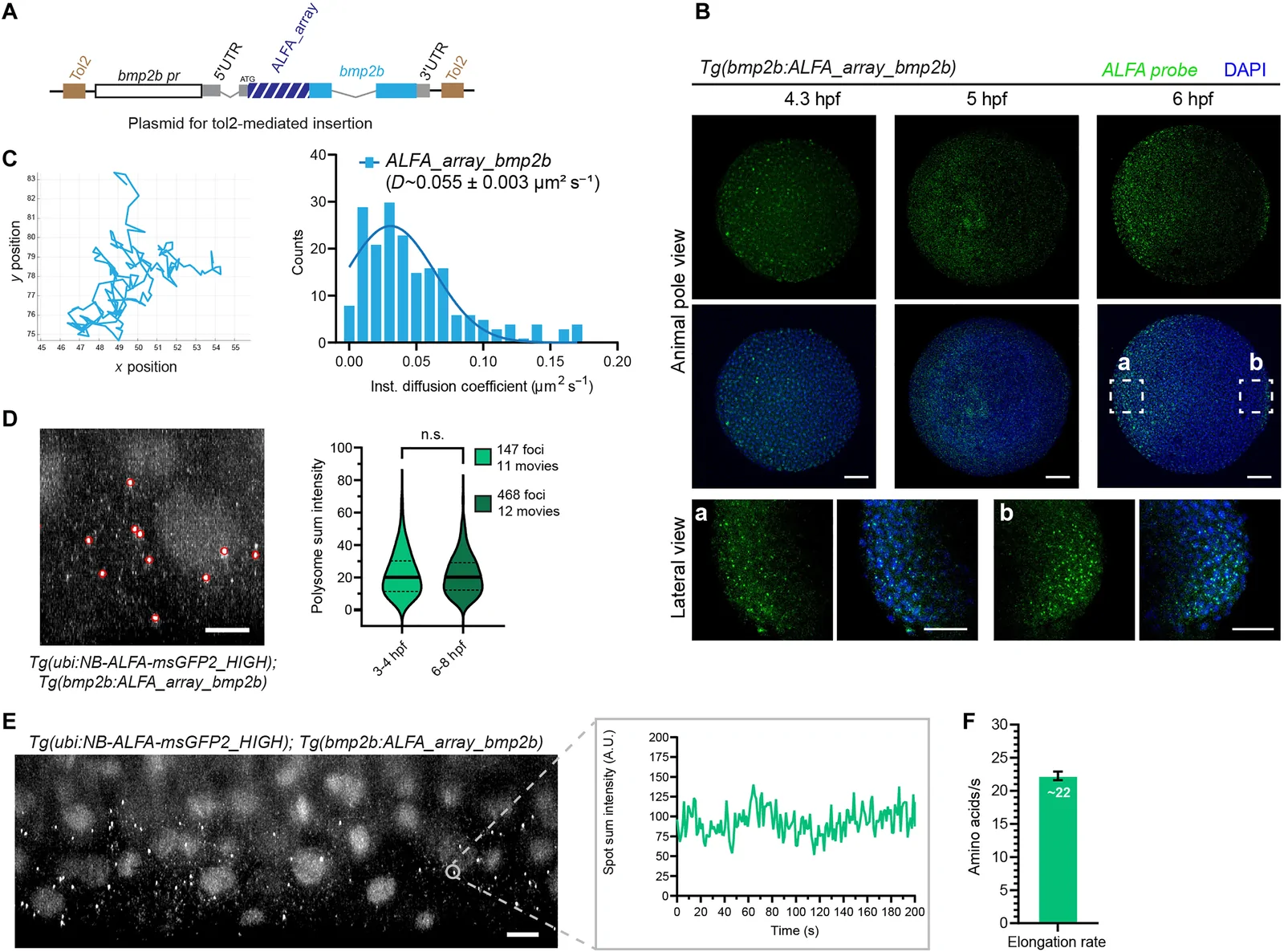
Tracking and quantifying *bmp2b* polysomes to determine translation kinetics. (**A**) Schematic of the *bmp2b* transgene construct containing the *ALFA_array* used for tol2-mediated insertion. (**B**) Images from fluorescence in situ hybridization using a probe against *ALFA_array* mRNA (green) in *Tg(bmp2b:ALFA_array_bmp2b)* embryos in animal pole view; ventral to the left. Bottom: Zoomed-in images show the transgene expression at 6 hours postfertilization in the ventral (a) and dorsal (b) side. (**C**) Left: Representative trajectory of a *bmp2b* mRNA particle in translation; *x* and *y* positions are in micrometers. Right: Frequency distribution of instantaneous diffusion coefficient of *bmp2b* mRNA molecules in translation in 3- to 4.3-hours postfertilization *Tg(ubi:NB-ALFA-msGFP2_HIGH); Tg(bmp2b:ALFA_array_bmp2b)* embryos (*n* = 259 traces, from 12 movies). *D* is the mean diffusion coefficient with the SEM. (**D**) Left: Representative maximum intensity–projected image of polysome detection (red circles) from an LLS movie of a ∼6-hours postfertilization *Tg(ubi:NB-ALFA-msGFP2_HIGH); Tg(bmp2b:ALFA_array_bmp2b)* embryo. Right: Polysome sum intensity quantification (see Materials and Methods) from 3- to 4.3-hours postfertilization (*n* = 147 from 11 movies) and 6- to 8-hours postfertilization (*n* = 468 from 12 movies) embryos. Black line represents the median; n.s., nonsignificant with unpaired *t* test (*P* value >0.05). (**E**) Left: Representative maximum intensity–projected image from an LLS movie of a ∼6-hours postfertilization *Tg(ubi:NB-ALFA-msGFP2_HIGH); Tg(bmp2b:ALFA_array_bmp2b)* embryo. Right: Example of the intensity over time of a tracked *bmp2b* polysome (gray circle and inset). (**F**) Mean values of the elongation rate of *ALFA_array_bmp2b* mRNA during translation (*n* = 43). The estimated parameter is indicated with the respective 95% confidence intervals. Scale bars, 100 μm (B), 50 μm [zoomed in (B)], 5 μm (D), and 10 μm (E). hpf, hours postfertilization.

We then analyzed published ribosome profiling and RNA sequencing datasets (*20*, *27*) and found that *bmp2b* belongs to a group of genes with low translation efficiency (fig. S4, D and E). To better understand the basis for this low efficiency, we sought to determine the dynamic features of *bmp2b* translation directly and in vivo. We first tracked individual translating *bmp2b* mRNAs (Fig. 3C), calculated their instantaneous diffusion coefficient, and compared *ALFA_array_bmp2b* expression originating from the injected plasmid with that from the stably integrated transgene. Polysome trajectories in both experimental conditions showed no preferential directional movement (fig. S5A), and mean square displacements (MSDs) as well as diffusion coefficients were comparable between mRNAs originating from the *ALFA_array*_*bmp2b* plasmid (fig. S5, B and C) and the transgene (Fig. 3C and fig. S5D), with an average diffusion rate of ∼0.055 μm^2^/s (Fig. 3C and fig. S5C), comparable to measurements reported for mRNA diffusion in invertebrate and mammalian systems (*32*, *37*). In addition, we quantified polysome intensities at single time points, which provides a readout of the number of nascent polypeptide chains on each mRNA. Since each actively translating ribosome contributes to the fluorescence signal of the ALFA_array, these intensities serve as a proxy for ribosome occupancy on the mRNA. Using this approach, we observed that, at the single-molecule level, *bmp2b* mRNAs were translated with similar efficiency both before and during gastrulation (3 to 4.3 hours postfertilization and 6 to 8 hours postfertilization, respectively; Fig. 3D), indicating stable ribosome loading across these developmental stages. Background fluorescence levels in translating cells remained unchanged (fig. S5E), indicating that the pool of free NB-ALFA remains stable between the analyzed time points in *Tg(bmp2b:ALFA_array_bmp2b)* embryos. Furthermore, we observed an increased number of foci per cell at 6 to 8 hours postfertilization compared with 3 to 4.3 hours postfertilization, reflecting the higher mRNA expression level at this stage, as measured by RNA sequencing (fig. S5F).

We next sought to determine the kinetics of translation on single mRNAs. We used LLSM to track individual polysome foci in 3- to 6-hours postfertilization embryos and extracted single-spot intensity fluctuations as a quantitative readout of translation dynamics (*33*, *37*, *56*) (Fig. 3E and movie S6). After applying photobleaching correction (see Materials and Methods and fig. S5G), as well as autocorrelation analysis and fitting (fig. S5, H to J), we estimated an average elongation rate of ∼22 amino acids per second Fig. 3F. While this elongation rate estimate may represent an upper bound and does not represent the heterogeneity between transcripts, it is within the range of previously reported rates in mammalian cells (from ∼3 to 22 amino acids per second) (*31**–**33*, *57**–**60*) and *Drosophila* embryos (∼5 and 35 amino acids per second) (*37*, *41*). Together, these findings establish the ALFA_array system as a physiologically relevant platform for real-time studies of translation dynamics in vertebrate embryos.

### The poly(A) tail promotes *bmp2b* translation with moderate impact on ribosome loading

In zebrafish embryos, the poly(A) tail is essential for posttranscriptional control, allowing maternal mRNAs to be properly stored, activated, and degraded in a tightly regulated developmental program (*61*, *62*). Although poly(A) tails are well known to enhance translation by promoting ribosome recruitment (*63*, *64*) and increasing mRNA stability (*62*), it remains difficult to distinguish between these two contributions in the context of a living embryo. Moreover, the relationship between poly(A) tail length and translation efficiency is context dependent and does not always show a direct correlation (*61*).

To investigate how the presence of a poly(A) tail influences *bmp2b* mRNA translation in the early zebrafish embryo, we compared by Western blot analysis the protein output following *bmp2b* (tagged with the ALFA_array) mRNA injection with and without a poly(A) tail. We observed that nonpolyadenylated *bmp2b* mRNAs yielded lower protein output than their polyadenylated counterparts (fig. S6A). To determine whether this reduction in protein output was due to a decreased amount of mRNA competent for translation or impaired translation efficiency, we generated a chimeric construct by replacing the *bmp2b* 3′UTR with the ZIKV 3′UTR (Fig. 4A). The ZIKV 3′UTR lacks a poly(A) tail, but its RNA escapes degradation because of its highly structured conformation (*65*, *66*), thereby allowing one to uncouple mRNA stability from poly(A)-dependent translation. We in vitro–transcribed mRNAs with either the *bmp2b* 3′UTR or the ZIKV 3′UTR, both without a poly(A) tail, and injected them into *Tg(ubi:NB-ALFA-msGFP2_HIGH)* embryos. LLS live imaging revealed that a higher proportion of mRNAs containing the ZIKV 3′UTR were engaged in translation (Fig. 4C) and that these mRNAs displayed higher polysome intensity, thereby resulting in increased protein output (fig. S6B). In contrast, *bmp2b* 3′UTR transcripts lacking a poly(A) tail displayed only a moderate decrease in polysome intensity compared with *bmp2b* 3′UTR with a poly(A) tail (Fig. 4B). Together, these results indicate that, for the *bmp2b* reporter, the poly(A) tail has only a modest effect on ribosome loading, suggesting that it primarily acts on the mRNA’s competence to be translated.

**Fig. 4.**
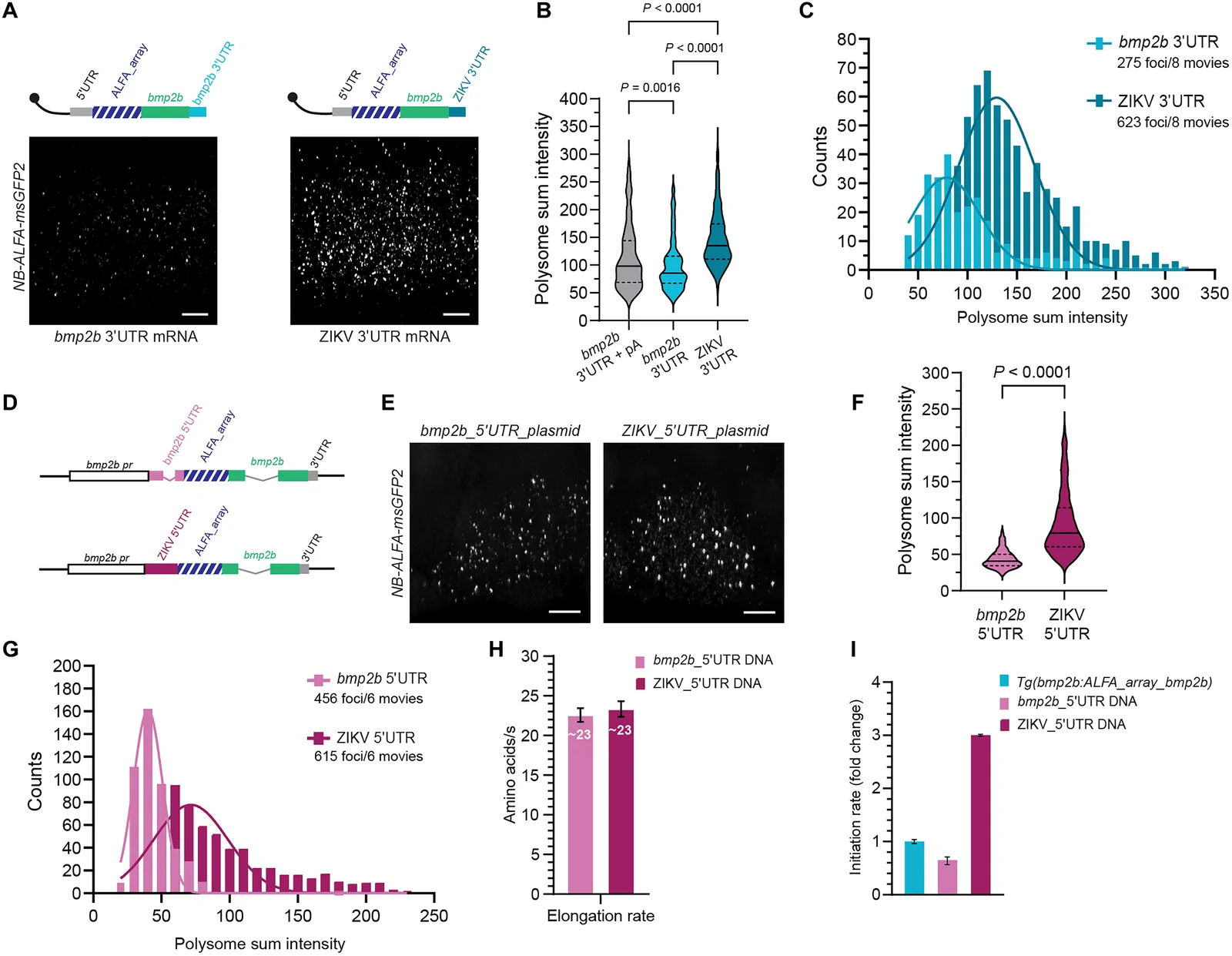
Comparative analysis of *bmp2b* and viral UTRs reveals *bmp2b* translation regulation in early zebrafish embryos. (**A**) Schematic of the in vitro–transcribed *ALFA_array_bmp2b* mRNA containing the *bmp2b* 3′UTR (light blue) or ZIKV 3′UTR (dark blue) without poly(A) tail. Representative maximum intensity–projected images from LLS movies of ∼1.5-hours postfertilization *Tg(ubi:NB-ALFA-msGFP2_HIGH)* embryos injected with the respective mRNAs. (**B** and **C**) Violin plot (B) and frequency distribution (C) of polysome sum intensity for *bmp2b* 3′UTR (*n* = 275 from eight movies) and ZIKV 3′UTR (*n* = 623 from eight movies) *ALFA_array_bmp2b* mRNAs without poly(A) tail. One-way analysis of variance (ANOVA); Gaussian fits are shown. (**D**) Schematic of *ALFA_array_bmp2b* DNA containing the *bmp2b* 5′UTR (pink) or ZIKV 5′UTR (dark pink). (**E**) Representative maximum intensity–projected images from LLS movies of ∼4.3-hours postfertilization *Tg(ubi:NB-ALFA-msGFP2_HIGH)* embryos injected with the *bmp2b* or ZIKV 5′UTR *ALFA_array_bmp2b* DNA. (**F**) Polysome sum intensity of *bmp2b* 5′UTR (*n* = 456 from six movies) and ZIKV 5′UTR (*n* = 615 from six movies) *ALFA_array_bmp2b* mRNAs. Two-tailed Welch’s *t* test. (**G**) Frequency distribution of polysome sum intensity from ∼1.5-hours postfertilization *Tg(ubi:NB-ALFA-msGFP2_HIGH)* embryos injected with the *bmp2b* or ZIKV 5′UTR *ALFA_array_bmp2b* DNA constructs. Gaussian fits are shown. (**H**) Mean elongation rates of *bmp2b* 5′UTR (*n* = 45 from six movies) and ZIKV 5′UTR (*n* = 51 from six movies) *ALFA_array_bmp2b* mRNAs, measured at ∼4.3 hours postfertilization. Parameters are shown with 95% confidence intervals. (**I**) Mean ribosome initiation rates of *ALFA_array_bmp2b* mRNAs from *Tg(bmp2b:ALFA_array_bmp2b)* embryos (*n* = 43 from 11 movies) and embryos injected with *bmp2b* 5′UTR (*n* = 45 from six movies) or ZIKV 5′UTR (*n* = 51 from six movies) *ALFA_array_bmp2b* DNA constructs, measured at ∼4.3 hours postfertilization in *Tg(ubi:NB-ALFA-msGFP2_HIGH)* embryos. Values are fold change relative to the initiation rate in *Tg(bmp2b:ALFA_array_bmp2b)* embryos, set to 1. Parameters are shown with 95% confidence intervals. Scale bars, 10 μm.

### Translation from the *bmp2b* 5′UTR is under initiation-level control

To directly assess the ribosome loading potential of the *bmp2b* mRNA, we substituted its 5′UTR with the ZIKV 5′UTR in our *ALFA_array_bmp2b* reporter, while keeping the coding sequence unchanged, enabling a direct comparison of their translation efficiency (Fig. 4D). We performed LLS imaging of *Tg(ubi:NB-ALFA-msGFP2_HIGH)* embryos following the injection of ALFA_array-tagged *bmp2b* plasmids containing either the *bmp2b* 5′UTR or the ZIKV 5′UTR under the same promoter, leading to the expression of endogenously capped and poly(A) tailed mRNAs (Fig. 4, D and E). We measured polysome intensities and observed that the ZIKV 5′UTR polysomes exhibit a higher intensity compared with the *bmp2b* 5′UTR polysomes (Fig. 4, F and G). Consistent with our single-molecule data, we observed increased protein output from the construct containing the ZIKV 5′UTR compared with the one containing the *bmp2b* 5′UTR (fig. S6C). We also observed increased protein output from in vitro–transcribed mRNAs when using the ZIKV 5′UTR (fig. S6D) compared with the *bmp2b* 5′UTR, indicating that the increase observed when injecting the plasmids is not due to differences in plasmid transcription levels.

Together, these data suggest that ZIKV 5′UTR mRNAs may be covered by more ribosomes than *bmp2b* 5′UTR mRNAs, potentially reflecting differences in ribosome initiation rates. To test this hypothesis, we tracked individual polysomes and quantified temporal fluctuations in fluorescence intensity for both *bmp2b* and ZIKV 5′UTR constructs. Autocorrelation analysis of these signals (fig. S6, E and F) allowed extraction of translation kinetic parameters (Fig. 4H). As expected, elongation rates were similar between constructs (Fig. 4H; also consistent with the transgene in Fig. 3F), whereas ribosome initiation rates were up to fivefold higher on ZIKV 5′UTR transcripts (Fig. 4I). These results indicate that, in the early embryonic context, the *bmp2b* 5′UTR does not drive translation at maximal capacity.

### The *bmp2b* 5′UTR enables noncanonical translation initiation in early zebrafish embryos

Given that it was described in cells in culture that the ZIKV 5′ and 3′UTRs contain highly structured RNA elements capable of promoting cap-independent translation (*29*, *67*), we hypothesized that the observed increase in translation efficiency was due to an IRES-like activity in its 5′UTR, independent of the rest of its genome. To test this hypothesis, we first in vitro–transcribed separately the *bmp2b* and ZIKV 5′UTR, fused to *bmp2b* coding sequence tagged with the ALFA_array (fig. S7A), with the nonfunctional cap analog A(ppp)G and a poly(A) tail and injected the resulting mRNAs into embryos for LLS imaging (Fig. 5A). In the absence of a functional cap, the ZIKV 5′UTR still drove robust polysome formation and translation (Fig. 5A), consistent with efficient cap-independent initiation. Furthermore, transcripts containing the ZIKV 5′UTR produced more protein (fig. S7B) and exhibited higher polysome intensity compared with those carrying the *bmp2b* 5′UTR (Fig. 5B) in the presence of cap analogs. Despite analyzing an equivalent number of imaging datasets, transcripts with the ZIKV 5′UTR also yielded a greater number of active polysomes (Fig. 5C), suggesting enhanced ribosome recruitment as well as increased mRNA engaged in translation. Injection of in vitro–transcribed mRNAs containing the *bmp2b* 5′UTR with the nonfunctional cap analog A(ppp)G led to a measurable level of translation (Fig. 5A and fig. S7C). This clearly detectable protein output from A(ppp)G-capped mRNAs further suggests that early zebrafish embryos can support noncanonical translation initiation. To assess specificity, we also injected *bmp2b* mRNAs bearing the *actin beta 2* (*actb2*) 5′UTR and capped with the same nonfunctional A(ppp)G. In this case, we did not detect substantial protein production (Fig. 5D); although with a functional cap, the *actb2* 5′UTR produced at least as much protein as the *bmp2b* 5′UTR (fig. S7D), suggesting that cap-independent translation is not a general feature of all 5′UTRs. Furthermore, to investigate the mode of cap-independent translation with *bmp2b* 5′UTR in the early zebrafish embryo, we performed LLS imaging of *bmp2b* reporter mRNAs carrying either the A(ppp)G- or G(ppp)G- cap (Fig. 5E). Because of extensive cell movements during early zebrafish embryogenesis (0.5 to 2 hours postfertilization), long-term tracking of individual mRNAs to extract translation kinetics was not feasible. Although polysome intensities were slightly higher for mRNA with the nonfunctional cap, the two transcripts were nearly identical (Fig. 5F), indicating no major difference in ribosome initiation rates for cap-dependent and cap-independent translation. Because polysome intensities were comparable between injected *ALFA_array_bmp2b* mRNAs and plasmid-derived transcripts (fig. S7E), the observed effects are unlikely to result from overexpression. Together, these findings indicate that early zebrafish embryos can sustain efficient translation with comparable initiation rates through both cap-dependent and cap-independent mechanisms, thereby revealing a gene-specific layer of translational regulation.

**Fig. 5.**
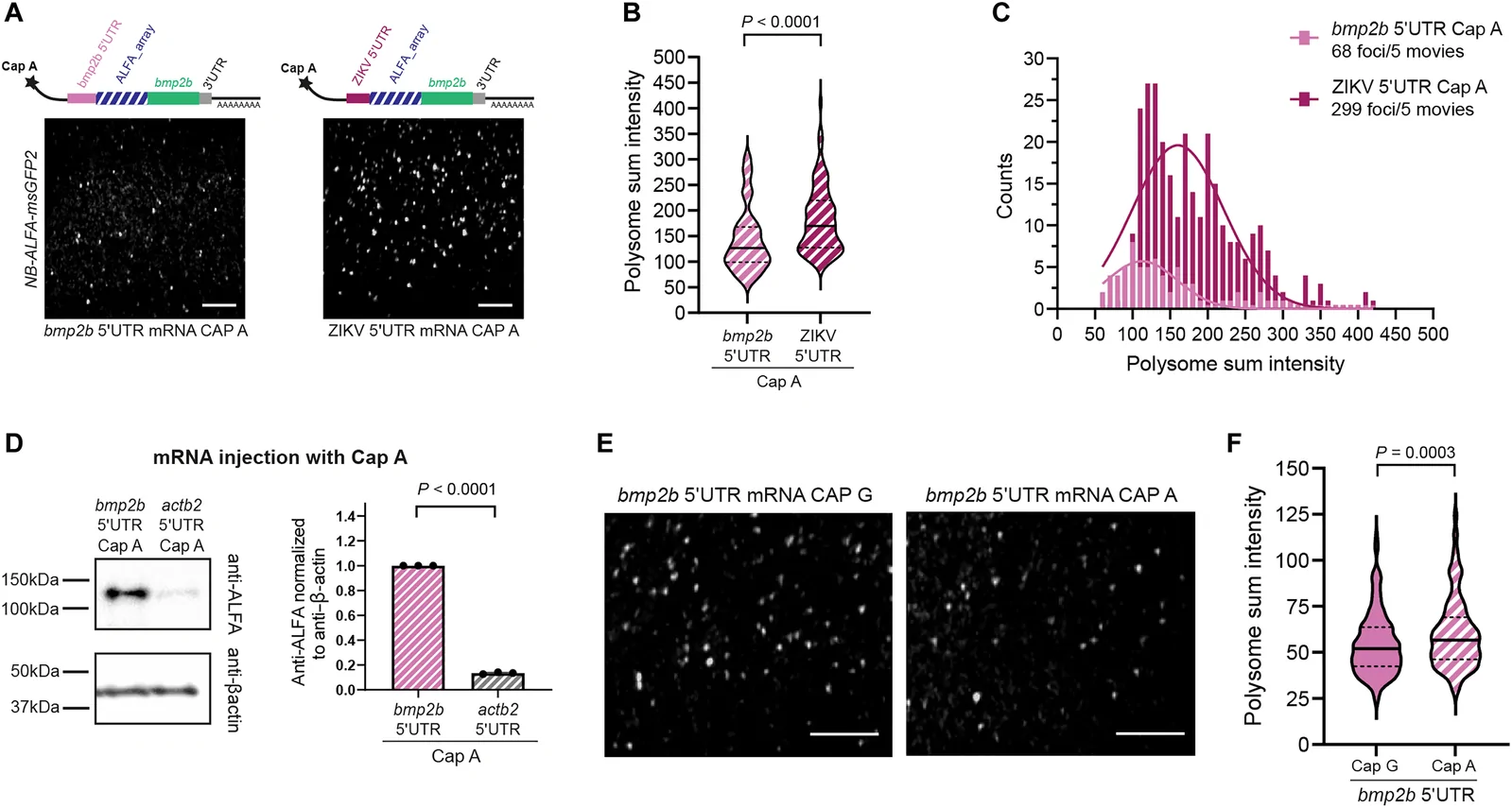
UTR-specific cap-independent translation initiation operates in early zebrafish embryos. (**A**) Schematic of the in vitro–transcribed mRNA of *ALFA_array_bmp2b* containing the *bmp2b* 5′UTR (pink) or ZIKV 5′UTR (dark pink) with the nonfunctional cap analog A(ppp)G. Maximum intensity–projected images from LLS movies of ∼1.5-hours postfertilization *Tg(ubi:NB-ALFA-msGFP2_HIGH)* embryos injected with the respective mRNA. (**B** and **C**) Violin plot (B) and frequency distribution (C) of polysome sum intensity of *bmp2b* 5′UTR (*n* = 68 from five movies) and ZIKV 5′UTR (*n* = 299 from five movies) *ALFA_array_bmp2b* mRNAs measured from the movies shown in (A). Unpaired *t* test was performed. (**D**) Western blot analysis, using anti-ALFA and anti–β-actin antibodies, of ∼1.5-hours postfertilization WT embryos injected with *bmp2b* 5′UTR or *actb2* 5′UTR *ALFA_array_bmp2b* mRNAs with an A(ppp)G (Cap A) cap (left); quantification from three independent crosses and injections (right). Two-tailed Welch’s *t* test was performed. (**E**) Maximum intensity–projected images from LLS movies of ∼1.5-hours postfertilization *Tg(ubi:NB-ALFA-msGFP2_HIGH)* embryos injected with *bmp2b* mRNAs of *ALFA_array_bmp2b* containing the *bmp2b* 5′UTR and capped with the functional Cap G or nonfunctional Cap A. (**F**) Violin plot of polysome sum intensity of *ALFA_array_bmp2b* mRNAs containing the *bmp2b* 5′UTR and capped with functional Cap G (*n* = 301 from 13 movies) or nonfunctional Cap A (*n* = 279 from 14 movies) measured from the movies shown in (E). Two-tailed Welch’s *t* test was performed. Scale bars, 10 μm.

## DISCUSSION

In this study, we present a framework for visualizing and quantifying translation dynamics in living vertebrate embryos by implementing the ALFA_array system to zebrafish. Using this approach combined with LLSM, we were able to directly visualize single *bmp2b* mRNA molecules in a living zebrafish embryo while simultaneously monitoring their translation at single-molecule resolution. Our data indicate that the low translation efficiency of *bmp2b* observed in bulk measurements (*20*, *27*) (fig. S4, D and E) primarily reflects regulation at the level of translation initiation and the limited fraction of mRNAs engaged in translation (Fig. 2E), rather than a reduced elongation rate, which itself falls within the range reported in other species (Fig. 3F) (*68*). Consistent with this, UTR switching with the ZIKV 5′UTR revealed that *bmp2b* translation does not operate at the maximal initiation capacity achievable in early zebrafish embryos (Fig. 4I). This observation suggests that the translational control of *bmp2b* relies mainly on the ability of individual mRNAs to enter translation and initiation rate modulation. We note that because imaging began before reliable embryo orientation (3 hours postfertilization) and since *bmp2b* mRNAs become dorsally enriched by 6 hours postfertilization, the quantified polysome signals and translation kinetics likely reflect predominantly dorsal translation. While our approach provides key insights into *bmp2b* translation dynamics, future investigation at the endogenous locus will help resolve its regulation within the full physiological context. We note that the N-terminal ALFA-array and associated nanobodies, once bound, may preferentially report cytoplasmic translation. Whether and to what extent endoplasmic reticulum–associated translation and subsequent secretory pathway processing contribute to the measured translation kinetics of *bmp2b* remains to be determined.

Removal of the poly(A) tail from the *bmp2b* 3′UTR had only a moderate effect on polysome intensity (Fig. 4B) but reduced protein production (fig. S6A). Therefore, in the context of 2-hours postfertilization zebrafish embryos, where poly(A) tail length has been shown to broadly modulate translational efficiency (*61*), the *bmp2b* poly(A) tail appears to support mRNA translatability rather than ribosome loading. In contrast, the *Orthoflavivirus* ZIKV 3′UTR restored stability and enhanced translation, even in the absence of a poly(A) tail, suggesting that it promotes translation initiation through specific structural or sequence elements. These findings support a role for the ZIKV 3′UTR as a poly(A)-independent translation enhancer, consistent with previous in vitro observations for other *Orthoflaviviruses* (*69*) but independent of other viral sequences.

Ribosome initiation rate is one of the main limiting factors in translation, and it is mostly driven by the 5′UTR (*24*, *25*, *27*). Our data reveal that substitution of the endogenous *bmp2b* 5′UTR with the ZIKV 5′UTR markedly enhanced translation efficiency at the single mRNA level. Notably, this enhancement persisted even in the absence of a functional cap, implicating the ZIKV 5′UTR in cap-independent translation, likely via an IRES-like mechanism. These data demonstrate in vivo that the ZIKV RNA can undergo cap-independent translation, reinforcing prior in vitro findings (*29*, *67*), and further show that such mechanisms can operate even without a viral 3′UTR, which is used in most studies (*29*, *67*). Together, our data highlight how viral UTRs can modulate translation, with the ZIKV 5′UTR efficiently hijacking the cellular machinery to maximize ribosome initiation and protein output, thereby enabling the virus to produce proteins as rapidly as possible, and surpassing the translational capacity of typical developmental genes.

In this study, we also show that an endogenous gene, *bmp2b*, is capable of cap-independent translation, uncovering a previously unrecognized mechanism regulating mRNA translation in the early vertebrate embryo. Notably, this mechanism appears to be specific to certain 5′UTRs, including that of *bmp2b*, as we did not observe similar cap-independent translation when testing the 5′UTR of the housekeeping gene *actb2* (Fig. 5D), suggesting that such regulation may be restricted to the 5′UTR of certain developmental genes. While the mechanism remains to be fully elucidated, our findings point to a cap-independent translation enhancer (CITE)–mediated mode of translation (*70**–**73*) or the presence of an IRES within the potentially highly structured *bmp2b* 5′UTR as suggested by secondary structure predictions (fig. S7, F and G). We found that cap-dependent and cap-independent mechanisms result in similar ribosome engagement in the early zebrafish embryo, consistent with a model in which cap-independent translation is not limited by the initiation rate, but rather by a reduced duration of the translationally active state as predicted in (*74*). In early zebrafish embryos (i.e., between 1 and 4 hours postfertilization), mechanistic target of rapamycin pathway markers of active canonical translation, including phospho-Rps6 and phospho–eIF4E-BP1, are reduced (*75*). This period of lower canonical translation may provide a window in which noncanonical, cap-independent translation is more permissive, consistent with our observation that *bmp2b* translation can occur efficiently at early embryonic stages. The early zebrafish embryo thus provides a valuable in vivo model to dissect noncanonical translation mechanisms and investigate the contributions of distinct 5′UTR elements to cap-independent gene expression.

In developmental or stress contexts where canonical cap- or poly(A)-dependent translation may be compromised, elements such as IRESs and CITEs can be co-opted to regulate and ensure robust endogenous expression. Noncanonical translation is particularly important in stress responses (*76*) and diseases such as cancer (*77*). Cap-independent translation also contributes to stem cell fate decisions via DAP5 (eIF4G2/NAT1) (*78*), which mediates the translation of specific genes in human embryonic stem cells (*79*). This noncanonical initiation factor is essential for embryonic development in *Drosophila* (*80*), mouse (*81*), and zebrafish (*82*), thereby highlighting a potentially critical role for cap-independent translation during embryonic development. Whether DAP5 contributes specifically to *bmp2b* translation in zebrafish remains an open question.

Together, our findings expand the toolkit for studying gene expression in vertebrates and also reveal how evolutionarily diverse UTRs modulate translation efficiency. Beyond *bmp2b*, whose expression has been well characterized, this platform allows single-molecule resolution analysis of translation in live zebrafish embryos. It opens the door to investigating translational regulation of other developmental genes, providing unprecedented spatiotemporal insights into how translation shapes cell fate, tissue patterning, and morphogen gradients.

## MATERIALS AND METHODS

### Zebrafish husbandry

Zebrafish husbandry was performed under institutional (MPG) and national (German) ethical and animal welfare regulations. Larvae were raised under standard conditions. Adult zebrafish were maintained in 3.5-liter tanks at a stock density of 8 zebrafish/liter with the following parameters: water temperature: 27° to 27.5°C; light/dark cycle: 14/10 hours; pH: 7.0 to 7.5; conductivity: 750 to 800 μS/cm. Zebrafish were fed three to five times a day, depending on age, with granular and live food (*Artemia salina*). Health monitoring was performed at least once a year. All embryos and larvae used in this study were raised at 28.5°C. All procedures performed on animals conform to the guidelines from Directive 2010/63/EU of the European Parliament on the protection of animals used for scientific purposes and were approved by the Animal Protection Committee (Tierschutzkommission) of the Regierungspräsidium Darmstadt (reference: B2/1218). Zebrafish housing and husbandry were approved by the “Comité d’Etique pour l’Expérimentation Animale et la Direction Sanitaire et Vétérinaire de l’Hérault” (Aquatic model facility ZEFIX CRBM, C-34-172-39).

### Animal experimentation

All experiments involving zebrafish were carried out on embryos/larvae before 5 dpf and complied with the guidelines of the European Parliament Directive 2010/63/EU on the protection of animals used for scientific purposes.

### Plasmid generation for zebrafish line establishment

The plasmids used to generate the *Tg(ubi:NB-ALFA-msGFP2_HIGH)bns698*, *Tg(ubi:NB-ALFA-msGFP2_LOW)bns699*, *Tg(ubi:scFv-msGFP2)bns715*, *Tg(ubi:NB-mScarlet3)bns740* lines were generated using NEBuilder HiFi DNA Assembly from published ubiquitin promoter (*45*), NB-msGFP2-GB1-NLS (*38*), and scFv-msGFP2-GB1-NLS (*38*) sequences in Tol2 plasmid containing eCFP under the control of gamma-crystallin promoter (a gift from D. Gilmour, Department of Molecular Life Sciences, University of Zurich). All three nanobodies were fused to a GB1 (B1 domain of streptococcal protein G) to improve solubility and to a nuclear localization signal (NLS) on the scFv and NB-msGFP2 to improve the signal-to-noise ratio in the cytoplasm. For the *Tg(bmp2b:ALFA_array_bmp2b)bns739, bmp2b* promoter [primer sequences from (*16*)], UTRs and coding sequence are from WT(AB) genomic DNA. The *ALFA_array* sequences from (*38*) were codon optimized for zebrafish (https://en.vectorbuilder.com/tool/codon-optimization.html) and synthesized at GenScript Biotech Corporation. The final plasmid was generated using NEBuilder HiFi DNA Assembly.

Transgenesis was done by injection of one-cell stage embryos with 20 pg of plasmid DNA together with 15 pg of transposase *Tol2* mRNA and 0,2% phenol red. The positive offspring were outcrossed with WT(AB) for two generations before establishing the stable line.

Full sequences obtained with whole plasmid sequencing are available in table S1.

### Plasmid generation for in vitro transcription

*bmp2b* UTRs and coding sequence were amplified from cDNA since it contains introns; the *ALFA_array* sequences were from (*38*), and a T7 sequence (TAATACGACTCACTATAGGG) was added and assembled using NEBuilder HiFi DNA Assembly. From this plasmid, 5′ or 3′UTR of *bmp2b* were removed, and 5′ (5′-AGTTGTTGATCTGTGTGAATCAGACTGCGACAGTTCGAGTTTGAAGCGAAAGCTAGCAACAGTATCAACAGGTTTTATTTTGGATTTGGAAACGAGAGTTTCTGGTC-3′) or 3′UTR (5′-GCACCAATCTTAATGTTGTCAGGCCTGCTAGTCAGCCACAGCTTGGGGAAAGCTGTGCAGCCTGTGACCCCCCCAGGAGAAGCTGGGAAACCAAGCCTATAGTCAGGCCGAGAACGCCATGGCACGGAAGAAGCCATGCTGCCTGTGAGCCCCTCAGAGGACACTGAGTCAAAAAACCCCACGCGCTTGGAGGCGCAGGATGGGAAAAGAAGGTGGCGACCTTCCCCACCCTTCAATCTGGGGCCTGAACTGGAGATCAGCTGTGGATCTCCAGAAGAGGGACTAGTGGTTAGAGGAGACCCCCCGGAAAACGCAAAACAGCATATTGACGCTGGGAAAGACCAGAGACTCCATGAGTTTCCACCACGCTGGCCGCCAGGCACAGATCGCCGAATAGCGGCGGCCGGTGTGGGGAAATCCATGGGTCT-3′) of Zika was amplified from a molecular clone of the BeH819015 isolate representative of the most prominent group of ZIKV isolates from South America as it differs very few from other sequenced isolates (*83*) and assembled using NEBuilder HiFi DNA Assembly.

The 30 MS2 loops were reengineered from (*84*) using prediction with (*85*) to obtain 30× functional, short, and low-affinity MS2 sequences and synthesized at GenScript Biotech Corporation and were added after the stop codon of *bmp2b*. Full sequences obtained with whole plasmid sequencing are available in table S1.

### In vitro transcription and injections of mRNAs

Capped mRNAs were synthesized using the HiScribe T7 ARCA mRNA Kit (New England Biolabs, no. E2065) following the manufacturer’s protocol. Poly(A) tailing was performed using an *E. coli* Poly(A) Polymerase kit (New England Biolabs, M0276). Purification of mRNAs was done with a Zymo Research RNA Clean & Concentrator kit (R1016).

For cap analog mRNA synthesis, a MEGAshortscript T7 Transcription kit (Invitrogen, AM1354) was used following the manufacturer’s protocol and adding the unmethylated G(5′)ppp(5′)A RNA Cap Structure Analog (New England Biolabs, no. S1406) or 3′-O-Me-m7G(5′)ppp(5′)G (New England Biolabs, no. S1411) in excess [2 mM each nucleoside triphosphate (NTP) and 8 mM cap analog].

For Western blot analysis, one-cell stage embryos were injected with 25 pg of mRNA in 1-nl volume of water. For lattice light-sheet imaging, one-cell stage embryos were injected with 2.5 pg of mRNA in 1-nl volume of water. For the puromycin experiment, 25 pg of mRNA in 1-nl volume of water with or without 10 mg/ml concentration of puromycin (Merck, P8833) was injected.

### Plasmid generation for transient experiments

*ALFA_array_bmp2b* plasmid was used from the generation of the transgenic line, and ZIKV 5′UTR (see the “plasmid generation for in vitro transcription” section for the sequence) was added using NEBuilder HiFi DNA Assembly.

For transient experiments, 20 pg of plasmid was injected without phenol red. All reagents were injected at a volume of 1 nl.

### Immuno-HCR-FISH

Immunostaining coupled with HCR-FISH (Molecular Instruments) was performed as described in (*86*) and according to the manufacturer’s protocol with few modifications. Embryos were fixed at the desired stage in 4% paraformaldehyde (PFA) for 16 hours at 4°C. Then, embryos were washed three times for 5 min each with 1 ml of phosphate-buffered saline (PBS). At that point, embryos were manually dechorionated using forceps. Embryos were incubated four times for 10 min each in methanol (MeOH) and once for 50 min. Embryos were stored at least overnight at −20°C in MeOH. Then, approximately nine embryos per tube were rehydrated with a series of MeOH/1× PBS–0.1% Tween 20 (PBST) washes for 5 min at room temperature (75% MeOH, 50% MeOH, 25% MeOH, and five times PBST). Embryos were prehybridized with 250 μl of hybridization solution (Molecular Instruments) for 30 min at 37°C and hybridized with 16 nM of probe in 250 μl of hybridization solution overnight at 37°C. Probes were designed by Molecular Instruments using the sequence of the ALFA_array (see table S1). The next day, embryos were washed three times for 15 min each with 250 μl of wash buffer (Molecular Instruments) at 37°C followed by two times for 5 min each in 500 μl of 5× SSC–0.1% Tween 20 (SSCT) at room temperature. Amplification step was done with a 30-min preamplification wash in 250 μl of amplification buffer (Molecular Instruments) followed by incubation in 250 μl of amplification buffer with 30 pmol of each hairpin [h1 and h2, HCR Amplifier (v3.0) 647 dye] overnight in the dark. Then, embryos were washed two times for 5 min each, two times for 30 min each, and once for 5 min in 5× SSCT. Then, the samples were washed three times for 5 min each in PBST and blocked with 0.5% ultrapure bovine serum albumin (Invitrogen, AM2616) in PBST for 2 hours at room temperature. Embryos were then incubated overnight at 4°C with rabbit anti-ALFA (1:1000, Nanotag Biotechnologies, N1581) in block solution. The next day, embryos were washed six times for 30 min each in PBST and incubated with 1/500e secondary antibody (goat anti-rabbit, Alexa Fluor 568, Thermo Fisher Scientific, A11036) overnight at 4°C. Then, embryos were washed six times for 30 min each in PBST and mounted in ProLong Diamond with 4′,6-diamidino-2-phenylindole (DAPI) (Invitrogen, P36962). Embryos were imaged on a Zeiss LSM800 Observer Airyscan.

### Whole-mount fluorescent in situ hybridization

The in situ probe for *ALFA_array* (of 800-nt length) was synthesized from a polymerase chain reaction product from the *bmp2b:ALFA_array_bmp2b* plasmid using the following primers: *ALFA_array*-ISH forward: 5′-GAAGCGGAAGCGGACCATCTAG-3′ and *ALFA_array*-ISH reverse: 5′ CAGATCAATTAACCCTCACTAAAGGGAGAAGGTCCACTTCCAGATC-3′. T3 RNA polymerase (Roche, catalog no. 11031163001) was used for transcription of probes using digoxigenin-labeled NTPs (Roche, catalog no. 11277073910). Adult *Tg(bmp2b:ALFA_array_bmp2b)* fish were intercrossed, and embryos were fixed at the desired time points in 4% PFA overnight at 4°C. Then, embryos were dehydrated through a graded MeOH series, with each step lasting 5 min: 25% MeOH, 50% MeOH, 75% MeOH, and 100% MeOH. They were subsequently stored at −20°C in MeOH until needed. On the first day, after rehydration and washing in PBS–0.1% Tween 20 (PBST), embryos were prehybridized in hybridization buffer [50% formamide, 5× SSC, 0.1% Tween 20, 9.2 mM citric acid, heparin (50 μg/ml), and tRNA (500 μg/ml)] at 70°C for 2 hours, then incubated overnight at 70°C with DIG-labeled RNA probe (1 ng/μl) in hybridization buffer. On the second day, embryos were subjected to a series of washes at 70°C, transitioning from 100% hybridization buffer to 2× SSC in 10-min steps (25, 50, 75, and 100% 2× SSC). This was followed by room-temperature washes in 75, 50, and 25% 0.2× SSC in maleic acid buffer (100 mM maleic acid and 150 mM NaCl, pH 7.5) containing 0.1% Tween 20 (MABT) for 10 min each, with a final 10 min wash in MABT. Embryos were then incubated in 2% blocking reagent (Roche, catalog no. 11096176001) in MABT for 1 hour, followed by overnight incubation at 4°C with a 1:2000 dilution of anti–digoxigenin-POD antibody (Roche, catalog no. 11207733910) in blocking buffer. On the third day, after additional washes (six times for 10 min each in PBST), embryos were stained using a 1:100 dilution of tyramide-fluorescein reagent in amplification buffer (Akoya Biosciences, NEL741001KT) for 30 min at room temperature. After washing in PBST three times for 10 min each, embryos were stained with DAPI and mounted in ProLong Diamond. Imaging was performed on a ZEISS Celldiscoverer 7 automated microscope equipped with an LSM 900 confocal module and an Airyscan 2 detector using a Plan-APOCHROMAT 20X / 0.95NA W objective.

### Light-sheet live imaging

Embryos were collected after laying and mounted in 0.75% low-melting agarose (Sigma-Aldrich, A4018) in a capillary immersed in egg water (Instant Ocean Aquarium Systems, with Milli-Q water). Embryos were imaged on a Light Sheet Z.1 with the following settings: 20×/1.0 Corr DIC M27 water objective, images (1920 × 1920 pixels) for msGFP2, a 488-nm laser was used, and 160z-positions were taken with 1-μm z-step (37.8 s per frame). For *Tg(ubi:NB-ALFA-msGFP2_HIGH)bns698* and *Tg(ubi:NB-ALFA-msGFP2_LOW)bns699* embryos with zygotic-only expression, images were acquired at 4-min intervals to accommodate longer recording durations and minimize data volume. Movies were maximum intensity projected and processed with Fiji (*87*).

### Fluorescence recovery after photobleaching

FRAP was performed in zebrafish embryos expressing NB-ALFA-msGFP2 that were injected with 20 pg of H2B_mCherry_ALFAtag plasmid (*51*) and imaged 1 hour later at 28.5°C. The following settings were used: a Zeiss LSM880 using a 40×/1.3 oil objective. Images (512 × 512 pixels, 16 bits per pixel, pixel size 0.104 μm, zoom 4×) were acquired every ∼6 s for 43 frames. Excitation: GFP with a laser at 488 nm and mCherry with a laser at 561 nm. Laser power was measured using the 10×/0.45 Plan-Apochromat Air objective and was ∼1.2 mW for 488 nm and ∼4.2 mW for 561 nm at 100% laser. For acquisition, the measured laser power was ∼60 μW (488 nm) and ∼83 μW (561 nm). A circular region of interest (ROI) (3-μm diameter) was bleached using a hundred pulses of 488 nm and 561 lasers at maximal power (100%) after four prebleach frames. To discard any source of fluorescence intensity recovery other than molecular diffusion, the measured fluorescence recovery in the bleached ROI region (*I*_bl_) was corrected by an unbleached ROI (*I*_unbl_) of the same nucleus and another ROI outside of the nuclei (*I*_out_) following the simple equation

$$\text{Iblcorr}\left(t\right)=\frac{\mathrm{Ibl}\left(t\right)-\text{Iout}\left(t\right)}{\text{Iunbl}\left(t\right)-\text{Iout}\left(t\right)}$$

### Confocal live imaging

Confocal live imaging was performed on a Zeiss LSM880 Airyscan with 40×/1.1 water objective. For NB-msGFP2 and scFv-msGFP2 imaging, we used the following settings: images (512 × 512 pixels, 488-nm laser, zoom × 2, 30 z-positions) were taken with 1.5-μm z-step and a pixel size of 0.28 μm (43.8 s per frame).

### Lattice light-sheet live imaging

After injection, embryos were manually dechorionated using forceps and mounted in 0.75% low-melting agarose (Sigma-Aldrich, A4018) with egg water in a Fluorodish (Dutscher, FD35-100). Embryos were imaged on a Lattice Light Sheet 7 (Zeiss LLS7) with the following settings:

For mRNA molecule imaging of *Tg(actb2:2xMCP-NLS-EGFP)* embryos injected with *ALFA_array_bmp2b_30XMS2*: A 488-nm laser was used with 20-ms exposure time and 2x-planes were taken with 0.5-μm x-step for 200 time series; the image size was 1024 × 512 pixels (180 ms per frame).

For simultaneous mRNA and translation imaging of *Tg(ubi:NB-ALFA-mScarlet3); Tg(actb2:2xMCP-NLS-EGFP)* embryos injected with *ALFA_array_bmp2b_30XMS2*: A 488-nm laser was used with 30-ms exposure time, a 561-nm laser was used with 30-ms exposure time, and 70x-planes were taken with 0.5-μm x-step for 10 time series; the image size was 478 × 2048 pixels (943 ms per frame).

For kinetics measurements of *Tg(bmp2b:ALFA_array_bmp2b)bns739*: A 488-nm laser was used at ∼265 μW (measured after the objective using a Thorlabs laser power meter). Images were acquired with an exposure time of 10 ms. A total of 70x-planes were collected with an x-step of 0.5 μm for each of the 180 time points. The image size was 674 × 2048 pixels and corresponds to an acquisition time of 1.19 s per frame.

For kinetics measurements of *Tg(ubi:NB-ALFA-msGFP2_HIGH)* embryos injected with *bmp2b* 5′UTR DNA or ZIKV 5′UTR DNA: A 488-nm laser was used at ∼265 μW (measured after the objective using a Thorlabs laser power meter). Images were acquired with an exposure time of 10 ms. A total of 70x-planes were collected with an x-step of 0.5 μm for each of the 180 time points. The image size was 674 × 2048 pixels and corresponds to an acquisition time of 1.19 s per frame.

For mRNA injections with/without puromycin: A 488-nm laser was used at ∼305 μW (measured after the objective using a Thorlabs laser power meter) with 10-ms exposure and a single x-plane; the image size was 674 × 2048 pixels.

For spot intensity analysis: A 488-nm laser was used at ∼305 μW (measured after the objective using a Thorlabs laser power meter) with 10-ms exposure time, and a 70x-plane was taken with a 0.5-μm x-step for 30 time series; the image size was 674 × 2048 pixels. Images were processed with the Zeiss Lattice Light Sheet processing tool using the “Deskew>Cover Glass Transfomation” tool from Zen Blue (2.3 lite) software.

### Kinetics measurement analysis

Movies for extraction of translation kinetics from *Tg(bmp2b:ALFA_array_bmp2b)bns739* or embryos injected with *bmp2b* 5′UTR DNA or ZIKV 5′UTR DNA were acquired on Lattice Light Sheet (Zeiss LLS7) and were cover glass transformed and maximum intensity projected with Zen Blue (2.3 lite) software. Fluorescence intensity fluctuations of translation sites were obtained using the spot detector in Fiji (*87*) plugin TrackMate LOG (Laplacian Of Gaussian), and tracking was done with the TrackMate Linear Assignment Problem (LAP) from Fiji plugins (*88*, *89*). To generate the tracked intensity fluctuation, each detected spot was divided by background at each time frame. Tracks were then selected if their time length was superior to 150 frames (178.5 s). The multiple-tau algorithm allows to avoid overweighting of the end of the correlation versus the beginning.

The elongation dwell time *T* was obtained by fitting the measured autocorrelation, with the following analytical expression as in (*33*, *34*, *37*)

$$\left(\tau \right)=\left(T-\tau \right)/\left(c.T^{2}\right).H\left(T-\tau \right)+G\infty$$

where *c* is the translation initiation rate and *H*(*t*) is the Heaviside step function equal to 1 for *t* > 0 and zero otherwise. We included a G∞ term in the fit as in (*37*), to account for a nonzero baseline at long correlation times, which can arise from instrumental offsets, background noise, or slow signal drifts. This prevents bias in the estimation of the correlation amplitude and the derived parameters such as the particle number. Autocorrelation curves were fitted with a Levenberg-Marquadt algorithm using Python.

### Polysome intensity measurements

Polysome intensity measurements were performed using ICY software (*90*). Movie files were imported into ICY, and the Spot Detector plugin was used with a manually defined threshold to identify objects. Only polysome intensities from the first frame were retained (to avoid photobleaching bias) for analysis. Background intensity was measured by selecting ROIs devoid of translation dots. For each movie, the mean background intensity was calculated from at least five ROIs. Last, each spot was divided by the respective value of the background. All graphs were generated using GraphPad Prism 10.2.3 (403). Outliers were removed using the ROUT method in GraphPad Prism with the lowest stringency (*Q* = 0.1%). Frequency distribution was done with a binning of 10. For statistical tests, first, an *F* test of equality of variances was performed. If the variances were equal, two-tailed unpaired *t* test was performed. If the variances were not equal, a two-tailed Welch’s *t* test was performed. In the violin plots, plain black lines represent the median and the dashed lines the quartiles.

### Western blot analysis

Embryos were collected at the indicated times postfertilization. For 1.5-, 3-, 4.3-, and 6-hours postfertilization embryos, the yolks were removed by hand with a scalpel. For 9-, 12-, and 24-hours postfertilization embryos, the yolks were removed using deyolking buffer (55 mM NaCl, 1.8 mM KCl, and 1.25 mM NaHCO_3_). Embryos were then snap-frozen in liquid nitrogen. Embryos were crushed with a pestle with 4 μl per embryo of 4× Laemmli Sample Buffer (Bio-Rad, 1610747) supplemented with 2-mercaptoethanol. Samples were then heated at 70°C for 10 min; the volume equivalent to 2.5 embryos per well was loaded on a 4 to 12% Bis-Tris NuPAGE gel (Invitrogen NP0335) and ran at 180 V for 1 hour. Protein transfer was done at 4°C for 1 hour at 110 V onto a 0.2-μm nitrocellulose membrane (Invitrogen, LC2000). The membrane was blocked in 5% milk-PBT (PBS + 0.1% Tween 20) for 40 min and incubated overnight at 4°C with rabbit anti-ALFA (1:1000, Nanotag Biotechnologies, N1581) or mouse anti–β-actin (1:1000, Sigma-Aldrich, A5441) primary antibodies in PBT. Anti-mouse (1:5000, Abcam, ab97023) and anti-rabbit (1:5000, Cell Signaling, 7074) horseradish peroxidase–linked secondary antibodies were used and incubated 1 hour at room temperature in 5% milk-PBT. Chemiluminescent detection was performed using the SuperSignal West Pico PLUS chemiluminescent substrate kit (Thermo Fisher Scientific, 34577) on a Bio-Rad ChemiDoc MP Imaging System. Signal quantification of Western blots was performed using Fiji (*87*) (mean intensity was taken for each band).

### MSD analysis

Movies were first cover glass transformed and maximum intensity projected with Zen software; single particles were detected using spot detector Fiji plugin TrackMate LOG, and tracking was done with the TrackMate LAP (*88*, *89*). In total, *n* = 2729 traces, from 8 movies for *bmp2b* 5′UTR construct and *n* = 3464 traces from 13 movies for ZIKV 5′UTR construct. One-dimensional displacement in *x* and *y* was obtained from TrackMate files from Fiji for each movie. MSDs were calculated for tracks present for at least 20 consecutive frames using the MSDanalyzer MATLAB script (*91*) on Matlab_R2024b software version. Instantaneous coefficients diffusion was obtained by fitting 0 to 20 frames of the MSD of individual trajectories with a linear model as follows

y=a*x + b

To estimate MSD, according to Einstein’s theory, the MSD of Brownian motion is described as

MSD(t) = 2dDt

with *d* representing dimensionality; in this case, *d* = 2. *D* represents the diffusion coefficient.

A linear fit was considered unsatisfactory when *R*^2^ < 0.8 and was removed from the dataset.
